# Positive Airway Cultures in Dogs and Cats Receiving Mechanical Ventilation for Tick Paralysis

**DOI:** 10.3390/ani12233304

**Published:** 2022-11-26

**Authors:** Suzanne Suk Kwan Tso, Ellie Leister, Claire Rebecca Sharp, Jane Heller, Justine S. Gibson

**Affiliations:** 1 Pet Intensive Care, Underwood, QLD 4119, Australia; 2 School of Veterinary Medicine, Murdoch University, Murdoch, WA 6150, Australia; 3 Heller Consulting Pty Ltd., Gulbali Research Institute, Charles Sturt University, Wagga Wagga, NSW 2678, Australia; 4 School of Veterinary Science, The University of Queensland, Gatton, QLD 4343, Australia

**Keywords:** aspiration pneumonia, ventilator-associated pneumonia, antimicrobials, susceptibility, hypoxaemia

## Abstract

**Simple Summary:**

Aspiration pneumonia is known to be a common complication of tick paralysis in dogs and cats. This study aims to describe the clinical course, and culture and susceptibility profiles, of dogs and cats mechanically ventilated for tick paralysis that had positive airway cultures. A retrospective electronic medical record review was conducted. Twenty-four dogs, and two cats were included. The majority of these cases also had concurrent evidence of aspiration pneumonia based on chest X-rays and arterial blood gas results. Appropriate use of antimicrobials was shown to improve outcome. Clinicians should have an index of suspicion for the development of bacterial pneumonia in dogs and cats undergoing mechanical ventilation for tick paralysis. Empirical antimicrobials appropriate for the likely organisms and based on illness severity should be commenced pending culture and susceptibility testing, after which antimicrobial escalation or de-escalation is indicated to optimise outcomes while adhering to principals of antimicrobial stewardship.

**Abstract:**

Animals with tick paralysis often require mechanical ventilation (MV) but previous publications have identified knowledge gaps regarding the development of bacterial pneumonia, and the specific pathogens involved. The objectives of this study were to describe the clinical course and culture and susceptibility profiles of bacteria isolated from airway samples of dogs and cats mechanically ventilated for tick paralysis that had positive airway cultures. Medical records were reviewed, and cases included if they had a positive airway sample culture during MV for tick paralysis. Twenty-four dogs and two cats were included. Most (85%) received empirical antimicrobials before airway sampling. The most common organisms isolated included *Staphylococcus* spp. (11), *Klebsiella* spp. (9), *Enterococcus faecalis* (8), *Escherichia coli* (6), *Enterococcus faecium* (3), *Pseudomonas aeruginosa* (4), and *Mycoplasma* spp. (3). Evidence of aspiration pneumonia was present in 22/25 (88%) cases that had thoracic radiographs performed. Seventy-seven percent of cases received antimicrobials to which the cultured bacteria were susceptible during hospitalisation. The median duration of MV was 4 days (range 1–10). Most (77%) survived to discharge, 19% were euthanised, and one died. In a multivariable logistic regression analysis it was identified that selection of antimicrobials to which the causative bacteria are susceptible was associated with survival to discharge (Odds ratio 45.8, *p* = 0.014; 95%CI 1.98–14,808), as was length of MV, with every day an animal is ventilated associated with a 4.7 times increased chance of survival (*p* = 0.015; 95% CI 1.21–78.4).

## 1. Introduction

Tick paralysis caused by the Australian paralysis tick (*Ixodes holocyclus*) is a neuromuscular disease that affects the skeletomuscular, respiratory, gastrointestinal, and cardiovascular systems [1,2,3,4,5]. Female ticks release toxins from their salivary glands when feeding that prevents acetylcholine release at the neuromuscular junction causing progressive muscle paralysis [1,2]. Respiratory failure is a serious life-threatening complication in patients with tick paralysis, the mechanisms of which include respiratory muscle paralysis leading to hypoventilation, upper airway obstruction due to paralysis of the larynx and pharynx, central respiratory depressive effects of the holocyclotoxin, and lung parenchymal disease including aspiration pneumonia [1]. Pharyngeal and laryngeal dysfunction, and megaoesophagus are major predisposing factors of aspiration pneumonia in dogs and cats with tick paralysis [2,3].

Treatment of tick paralysis involves early administration of tick antiserum (TAS), intensive supportive care, and close monitoring of respiratory function [2,4,5,6,7,8,9]. Intubation and mechanical ventilation (MV) are indicated when there is severe hypercapnia, hypoxaemia unresponsive to the administration of supplemental oxygen, or unsustainable work of breathing, such that the patient is at risk of fatiguing to the point of respiratory arrest [2]. It has been previously demonstrated that tick paralysis cases that require MV due to oxygenation failure (lung disease) have significantly reduced survival (52.6%) compared to patients who required MV because of hypoventilation (90.5%) [7].

Bacterial pneumonia is one of the most likely causes of oxygenation failure in dogs receiving MV for tick paralysis. Aspiration pneumonia refers to the pulmonary bacterial infection that develops after aspiration [10]. There are no consensus definitions of aspiration pneumonia in veterinary medicine, rather the diagnosis is usually presumptive based on a combination of consistent history, clinical signs, and thoracic radiographic findings, with or without documentation of bacterial infection by airway culture [11]. Patients undergoing MV are also at risk of ventilator-associated pneumonia (VAP), that is, pneumonia that arises or acutely progresses after more than 48 h of endotracheal intubation and ventilation [12]. Although pneumonia is recognised to complicate the clinical course of small animals with tick paralysis, particularly those requiring MV, previous publications have identified knowledge gaps regarding the development of pneumonia and the specific pathogens involved [7]. Therefore, it is important to accurately describe the subgroup of patients that experience pneumonia during MV for tick paralysis. 

The objectives of this study were to describe the clinical course and culture and susceptibility profiles of bacteria isolated from airway samples of dogs and cats mechanically ventilated for tick paralysis that had positive airway cultures. We hypothesised that cases that received inappropriate antimicrobials would require a longer duration of mechanical ventilation and be less likely to survive than those receiving appropriate antimicrobials. 

## 2. Materials and Methods

Data were collected retrospectively from the medical records of a privatively owned intensive care unit (Pet ICU) within a specialist referral and emergency practice in South-eastern Queensland between January 2015 and February 2022. Patient records of a single visit that contained the following billing items “ventilator set up fee” or “hospital level 4” and “culture swab” or “body fluid culture” were cross searched to identify cases that were mechanically ventilated and had a positive airway culture. Retrieved records were examined to determine if they met the inclusion criteria. Cases were included if a patient had a record of a positive tick search (i.e., finding an engorged tick or crater), required MV due to tick paralysis, and had a positive bacterial culture from an airway sample while mechanically ventilated. Data including signalment (age, sex, breed), season, date of commencing MV, duration of ventilation, TAS administration and dosage, antimicrobial usage, and outcome were retrieved from the medical records. Information pertaining to airway culture was obtained, including the method of sample collection (bronchoalveolar lavage [BAL] or endotracheal aspirate [ETA]), the timing of sampling before or after antimicrobial use, bacterial isolates identified, susceptibility profiles, and change of antimicrobials after culture and susceptibility results. Additional details of the clinical course, including radiographic evidence of aspiration, specific lung function markers (ratio of partial pressure of oxygen in the arterial circulation [PaO_2_] to fraction of inspired oxygen [FiO_2_][P:F ratio], oxygen saturation [SpO_2_]), and outcome were also recorded. A diagnosis of aspiration pneumonia was made from a positive airway culture together with a clinical course of worsening lung function, indicated by hypoxaemia (P:F ratio ≤ 350 [13], or SpO_2_ < 95% with FiO_2_ ≥ 0.21), with or without thoracic radiographic evidence of aspiration pneumonia [14,15,16]. At the time of initial retrospective data collection the primary author (ST) interpreted all thoracic radiographs. Post hoc, available radiographs were also sent to a teleradiology provider (VetCT, https://www.vet-ct.com/au/, accessed on 14 November 2022.) for interpretation by a single board certified radiologist blinded to clinical data but informed of the study objectives and inclusion criteria. Patient outcome was classified as survival to discharge, natural death, or euthanasia. If a patient was euthanised, the main cause of euthanasia was noted when present. 

All cultures were performed by a single external laboratory (Queensland Medical Laboratory, Sunnybank Hills, QLD, Australia). Susceptibility data were presented as either “sensitive” or “resistant”. Additionally, regardless of the laboratory reporting, organisms were described as having intrinsic resistance (IR) if denoted as such by the Clinical and Laboratory Standards Institute (CLSI) VET01S guidelines [17]. Patients were classified as receiving an appropriate antimicrobial if the organisms from the airway culture were susceptible to either the initial antimicrobial regimen or if the patient was changed to an antimicrobial classified as susceptible. This definition of appropriate antimicrobial (i.e., With activity in vitro against the causative pathogens) was based on that used in the human sepsis and critical care literature [18,19], however, does not consider antimicrobial importance. As such, antimicrobials were also classified according to importance based on Australian Strategic and Technical Advisory Group on Antimicrobial Resistance (ASTAG) guidelines [14,15,16]. Multidrug resistant (MDR) organisms were defined as those not susceptible to at least one agent in three or more classes of antimicrobials to which they are usually susceptible [17]. 

Categorical data were summarised as proportions. Given the anticipated small sample size, continuous data were summarised as median and range. Univariable analyses were performed using Fisher’s exact tests for categorical data and Wilcoxon rank sum test for continuous data to assess the effect of the use of antimicrobials to which the identified organisms were susceptible (Yes/no), number of days ventilated, and presence of MDR organisms (Yes/no) on survival to discharge. A multivariable logistic regression analysis was subsequently performed including all three explanatory variables. Significance was set at *p* < 0.05. 

## 3. Results

The search strategy identified 26 cases, including 24 dogs and two cats that had a positive airway culture during MV for tick paralysis. Age ranged from four months to 13 years (median five years). Sex distribution was as follows: 10 desexed males, seven entire males, five desexed females, and four entire females. The most common dog breeds were poodle or poodle cross (4/24, 17%), Maltese or Maltese cross (3/24, 12%), and Pomeranian (3/24, 12%), followed by border collie and border collie cross (2/24, 8%), Cavalier King Charles spaniel (2/24, 8%), and lhasa apso (2/24, 8%). Eight other breeds were represented once. The two cats were a Ragdoll and Maine coon. 

Most patients (19/26, 73%) were admitted in Spring (September to November), four (4/26, 15%) were admitted in Summer (December to February), and three (3/26, 12%) were admitted in Winter (June to August). Tick locations were reported in 18/26 (69%), with most in the head and neck area (14/18, 78%). If an engorged tick was identified, it was immediately removed. All patients received intravenous TAS of varying dosages either at the authors’ hospital (21/26, 80.8%, which included both cats) or at the referring practice (5/26, 19.2%). Information about TAS dosing was identified for 20/21 cases that received TAS at the authors’ hospital. The standardised TAS protocol for dogs was 1 mL/kg or 20 mL per dog IV, whichever is the larger volume. The median TAS dose for dogs was 1.4 mL/kg (range 1–3 mL/kg), or 15.75 mL total dose (range 10–43 mL). Both cats received 5 mL of TAS IV. Information about TAS dosing was available for 2/5 cases that received TAS at the referring practice; the doses were 1 mL/kg and 1.2 mL/kg. All patients received an acaricide registered for tick paralysis to prevent ongoing intoxication and tick searches were performed every four hours throughout hospitalisation to ensure no further ticks were present. Other supportive treatments including oxygen supplementation, intravenous fluid therapy, anxiolytics, anti-emetics, and antacids were provided based on individual clinicians’ discretion. 

Most cases were mechanically ventilated on the day of admission 16/26 (62%), while the remainder 10/26 (39%) commenced MV the day after admission. The primary reason for intubation and MV was unsustainable work of breathing (21/26, 80.8%), followed by upper airway obstruction (3/26, 11.5%), hypoxaemia (1/26, 3.8%), and following successful cardiopulmonary arrest (1/26, 3.8%). Five cases (5/26, 19.2%) were identified to be hypoxemic immediately after intubation. Most cases (22/26, 85%) received antimicrobials before airway sampling was performed, while 4/26 (15%) had airway sampling prior to any antimicrobial usage. Of those that received antimicrobials before an airway sample, 14/22 (64%) received a single agent including cephazolin (*n* = 10), amoxicillin (*n* = 3), or amoxicillin-clavulanate (*n* = 1). Some dogs received a combination of cephazolin, and enrofloxacin (*n* = 2), cephazolin and metronidazole (*n* = 1), or cephazolin, metronidazole and enrofloxacin (*n* = 1). Others received a combination of ampicillin and enrofloxacin (*n* = 1), ampicillin, enrofloxacin and metronidazole (*n* = 1), or amoxicillin, enrofloxacin, and metronidazole (*n* = 1). Additionally, one dog received a combination of IV cephazolin and subcutaneous amoxicillin-clavulanate. One of the dogs that received cephazolin and enrofloxacin was later switched to piperacillin-tazobactam prior to airway culture due to clinical deterioration. Antimicrobial dose rates used in these cases were 22 mg/kg cephazolin IV q8h, ampicillin 20–22 mg/kg IV q8h, amoxicillin 20–22 mg/kg IV q8h, amoxicillin-clavulanate 15 mg/kg subcutaneous q24h, enrofloxacin 5 mg/kg IV q24h (2 cases) or 10 mg/kg IV q24h (4 cases), metronidazole 10 mg/kg IV q12h, and piperacillin-tazobactam 100 mg/kg IV q12h. Doses used were based on individual clinicians’ discretion. Based on the antimicrobial combinations reported above, three cases received a low importance antimicrobial (amoxicillin) according to the ASTAG importance ratings, 13 cases received at least one antimicrobial of medium importance (cephazolin, amoxicillin-clavulanate, or metronidazole), and 6 received at least one antimicrobial of high importance (enrofloxacin, piperacillin-tazobactam) prior to airway sampling. 

The time between the commencement of MV and airway sampling ranged from 0 to 4 days, with a median of 2 days. Most cases had ETAs performed (16/26, 62%), the remainder had BALs (10/26, 38%). 10/26 (38%) cases had a single bacterial isolate cultured. *Enterococcus faecium* was the most common single isolate cultured (2/10, 20%). Another 10 cases cultured two isolates, while 6/26 (23%) cultured more than three isolates. In total, 56 bacterial isolates and one fungal organism (*Candida* sp.) were cultured (Table 1). Three cases had positive airway cultures from two separate time points during MV, making a total of 29 positive cultures from 26 cases.

Table 2 displays antimicrobial susceptibility profiles of the cultured organisms, reporting proportions and percentages of isolates considered susceptible to given antimicrobials. No culture results reported intermediate susceptibility. Thirty six percent (18/56) of the cultured bacteria demonstrated MDR, and these were isolated from 16 animals. Two of the resistant *Staphylococcus pseudintermedius* isolates were oxacillin resistant and hence considered methicillin resistant *S. pseudintermedius* (MRSP). Extended susceptibility testing of these two isolates revealed susceptibility to nitrofurantoin and fusidic acid, one was susceptible to minocycline while the other was resistant, and both MRSP isolates were resistant to chloramphenicol and pradofloxacin. Both cases that cultured MRSP had received antimicrobials prior to their positive airway cultures. One case had received cephazolin and enrofloxacin (5 mg/kg IV q24h) for two days, followed by piperacillin-tazobactam for two days before airway sampling cultured MRSP. The second case received ampicillin, enrofloxacin (10 mg/kg IV q24h) and metronidazole IV for four days prior to culturing MRSP.

For the three cases with multiple positive cultures, the pattern of culture results varied. Case one had two ETA cultures submitted two days apart. The first cultured *Mycoplasma* species, and the second a coagulase negative *Staphylococcus* species. Case two initially had a BAL culture, followed by an ETA culture two days later. *Enterobacter aerogenes* with identical susceptibility profiles was cultured on both occasions. Additional isolates were a *Staphylococcus schleferi* and Group G *Streptococcus* from the BAL, and isolates of *Enterococcus faecalis* and *E. coli* from the ETA. Case three had two ETAs performed 6 days apart. Both ETAs cultured an isolate from the *Enterobacter cloacae* complex and *Pseudomonas aeruginosa* that where initially susceptible to enrofloxacin and later resistant. Additional isolates from the first culture in this case included *Klebsiella pneumonia* and *Staphylococcus pseudintermedius*.

Thoracic radiographs were performed in 25/26 (96%) cases. 24 cases had radiographs performed at the authors’ hospital (and subsequently reviewed by one of the authors), and one case had radiographs performed at the referring practice. Additionally, 10 thoracic radiographic series were sent to an external teleradiology company for review by a board-certified radiologist. Most (12/24, 50%) thoracic radiographs were taken on the day of intubation. Radiographs were taken one day prior to commencement of MV in 2/24 (8.3%), two days after MV in 3/24 (12.5%), and 3 or more days after commencing MV in the remainder (5/24, 20.9%). Radiographic evidence of aspiration pneumonia was present in 22/25 (88%) cases, while 3/25 (12%) had no pulmonary pathology evident on thoracic radiographs. The most common radiographic finding was an alveolar pattern in the right middle lung lobe (17/22, 77%), and most animals with evidence of aspiration pneumonia had more than one lung lobe affected (15/22 [68%]). In 5 cases there was one or more lung lobes with decreased volume concurrent with an alveolar pattern such that atelectasis could not be ruled out. Of the cases with no pulmonary pathology, two had radiographs taken on the day of intubation and one had radiographs taken 3 days after commencing MV.

Four dogs had serial thoracic radiographs performed, all of which survived to discharge. One of these dogs had radiographs taken on the day of commencement MV which did not reveal any pulmonary pathology. On the third day of MV the dog was witnessed to regurgitate, and an airway sample was collected for culture. On the fourth day after commencement of MV repeat thoracic radiographs revealed consolidation of all right lung lobes. The second dog had evidence of aspiration pneumonia (consolidated right middle and cranial lung lobes) on the day after commencement of MV, and this pulmonary pathology had resolved by the time of recheck thoracic radiographs 10 days later. The third dog had radiographs taken on the day of commencement of MV, identifying an alveolar pattern in the left cranial lung lobe. Two days after commencement of MV repeat radiographs revealed improvement of the left cranial lung lobe changes, with a new alveolar pattern in the right cranial and middle lung lobes. The fourth dog had an alveolar pattern in the ventral aspect of the left lung lobes on the day of commencement of MV, with resolution of the alveolar pattern, but a persistent bronchointerstitial pattern two days later, consistent with resolving pneumonia.

Arterial blood gases were performed in 20/26 (77%) patients during MV. All 20 cases had a P:F ratio of ≤350 with a median value of 212.5 (range 94–350). Specifically, the P:F ratio was <100 in 1/20 (5%), 100–200 in 8/20 (40%), 200–300 in 8/20 (40%), and 300–350 in 3/20 (15%). Five animals were identified to have hypoxaemia during MV based on SpO_2_ measurement alone, such that overall 25/26 patients (96%) were hypoxaemic during MV. Overall, two dogs did not fulfil our criteria for aspiration pneumonia. One dog with a positive airway culture (*Mycoplasma* sp. on day 4) lacked radiographic evidence of pneumonia but was hypoxaemic, while another with a positive airway culture (*Enterococcus* spp. and *E. coli*) had radiographic evidence of pneumonia without documentation of hypoxaemia during MV.

To differentiate cases of aspiration pneumonia from those that developed VAP, all cases that had a positive culture more than two days after intubation had their thoracic radiographs and blood gas results reviewed. Nine cases had a positive culture more than two days after intubation. Of these nine cases, five had thoracic radiographic evidence of aspiration pneumonia on the day of intubation, while three cases had radiographic evidence of aspiration pneumonia one or two day(s) after intubation. In addition, among these nine cases, 6/9 (78%) had at least one P:F ratio < 350 on either day zero or day one after intubation. Only one case met our criteria for VAP; this cat presented with severe respiratory distress characterised by open mouth breathing and cyanosis. It was intubated and ventilated immediately upon presentation. There was no evidence of pneumonia on thoracic radiographs on the day of intubation, but a positive airway culture on the third day after intubation, and radiographs consistent with pneumonia on the fourth day after intubation. Thus, with this case re-classified as VAP, 23 cases fulfilled the criteria for aspiration pneumonia, one for VAP, and two did not meet the criteria for either aspiration pneumonia or VAP.

The majority of patients, 22/26 (81%) had a change of antimicrobials during MV. Where the rationale for the change of antimicrobials was reported (13/20), the common causes were witnessed regurgitation (5/13), presence of new microbes on in-house ETA cytology (4/13), increase patient/ventilator dssynchrony (2/13), or hypoxaemia (2/13). No cases had de-escalation of antimicrobial therapy during hospitalisation.

Only 4/26 (15%) cases cultured microbes that demonstrated in vitro susceptibility to the initial antimicrobials prescribed. Another 12/26 (46%) cases cultured microbes that were resistant to the initial antimicrobials prescribed but were sensitive to the change of antimicrobial therapy, although the timing relative to receipt of culture results was difficult to ascertain. Six cases (6/26, 30%) cultured microbes that were resistant to all antimicrobials administered during hospitalisation, while the remaining four cases received antimicrobials that were not assessed in susceptibility testing. The six cases that did not receive appropriate antimicrobials cultured a *P. aeruginosa* isolate treated with amoxicillin-clavulanate, three MDR *E. faecium*, an *E. faecalis*, and an *Enterobacter* sp. all of which were treated with enrofloxacin to which they were resistant. The four cases that received antimicrobials not assessed in susceptibility testing included two that were considered to have received appropriate antimicrobials (Piperacillin-tazobactam for *E. faecalis*) and two classified as inappropriate (Meropenem for MRSP, and MDR *Staphylococcus haemolyticus* expected to be carbapenem resistant, based on resistance to all beta-lactams and cephalosporins tested, although not assessed for oxacillin resistance). Thus overall, 18/26 cases (69%) were considered to have received an appropriate antimicrobial during hospitalisation.

Regarding outcome, 20/26 (77%) cases survived to discharge, 5/26 were euthanised, and one died with unsuccessful cardiopulmonary resuscitation. The two cats survived to discharge. All patients that survived to discharge were still alive at the 30 day follow-up. Of the five patients euthanised, three were euthanised due to prognosis, including two that had recurrent episodes of regurgitation and suspected aspiration. The rationale for euthanasia was not described in the medical records of the two remaining cases and these were omitted from survival analysis. When considering outcome relative to antimicrobial appropriateness, 15/16 (93.8%) cases that received appropriate antimicrobials survived to discharge, compared to 1/5 (20%) that did not receive appropriate antimicrobials (*p* = 0.028).

Further consideration was given as to why some cases did not receive appropriate antimicrobials at any time during hospitalisation. The four non-survivors that did not receive appropriate antimicrobials died (1) or were euthanised (3) before culture and susceptibility results became available. The four survivors that did not receive appropriate antimicrobials all displayed marked clinical improvement between the time of sample collection and obtaining their culture results, such that the clinicians elected not to adjust their antimicrobial therapy.

**Figure 1:**
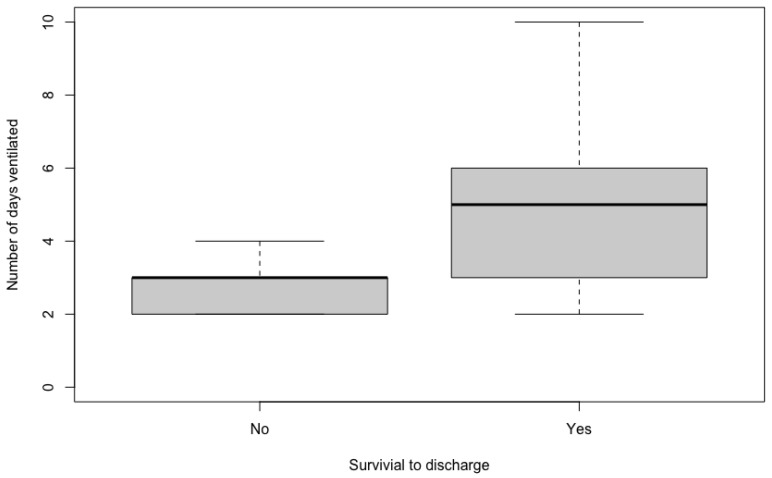
Box and whisker plot comparing the number of days of mechanical ventilation between survivors and non-survivors.

The duration of MV ranged from two to 10 days, with a median of four days. On univariable analyses, there was no significant difference between the duration of MV for cases that received appropriate antimicrobials (median 4.5 days, range 2–10), compared to cases that did not (median 4 days, range 2–6) (*p* = 0.475). Patients that survived to discharge had a significantly longer duration of ventilation (median 5 days, range 2–10), than nonsurvivors (median 3 days, range 2–4, *p* = 0.047) (Figure 1). The presence of MDR organisms was not associated with survival (*p* = 1).

The multivariable model identified that, independent of length of MV and presence of MDR organisms, on average, animals receiving appropriate antimicrobials have 45.8 times the odds of survival to discharge (*p* = 0.014; 95% CI 1.98–14,808), compared to those that did not receive appropriate antimicrobials. Furthermore, for each additional day of ventilation, odds of survival increase by 4.7 times (*p* = 0.015; 95% CI 1.21–78.4). There was no independent effect of the presence of MDR organisms on survival (*p* = 0.978).

## 4. Discussion

Herein we describe the clinical course and culture and susceptibility profiles of 24 dogs and two cats mechanically ventilated for tick paralysis that had positive airway cultures. It was identified that selection of antimicrobials to which the causative bacteria are susceptible was associated with survival to discharge, as was length of ventilation, with every day an animal is ventilated associated with an increased chance of survival.

The breeds represented, seasonal distribution, and tick locations reported in this study are consistent with the described epidemiology of tick paralysis in Eastern Australia [5,20]. Overall survival to discharge and duration of MV, although numerically greater (survival 77% vs. 63.9%, duration of MV median 4 days vs. 23 h), were in line with previous estimates from a study in the same hospital (63.9%) during an earlier time period [7], when considering the small numbers of dogs included in both studies. The association of longer duration of MV with improved survival suggests that if owners are able to afford the cost of care, good outcomes can be achieved despite longer periods of MV, even in patients that develop bacterial pneumonia.

The timing of initiation of MV in this study provides useful information for clinicians managing cases of tick paralysis. Since most patients commenced MV on the day of or day after admission the first 48 h of hospitalisation likely represents the highest risk period for respiratory complications of tick paralysis. The majority of patients in our case series received empirical antimicrobials prior to airway sampling, with cephazolin the most used antimicrobial. Although the rationale for the commencement of antimicrobials was not always documented, this suggests a high index of suspicion for bacterial pneumonia as a contributing factor to respiratory failure in these cases. The use of empirical antimicrobials in this case series is consistent with the Antimicrobial use Guidelines for Treatment of Respiratory Tract Disease in Dogs and Cats published by the Antimicrobial Guidelines Working Group of the International Society for Companion Animal Infectious Diseases (ISCAID) [21], who recommend that antimicrobial treatment should be initiated as soon as possible and empirical antimicrobial treatment should not be delayed in an effort to stabilise affected animals and obtain a pre-antimicrobial airway sample [15]. These guidelines recommend parenteral administration of a beta-lactam antimicrobial, such as amoxicillin or cephazolin, is sufficient in acutely affected animals without evidence of sepsis [15]. Although empirical cephazolin was administered more commonly than amoxicillin in this case series, amoxicillin could be considered a more appropriate first choice from the perspective of antimicrobial stewardship. According to ASTAG guidelines, amoxicillin is categorised as low importance for the mitigation of antimicrobial resistance, since there are a reasonable number of alternative antimicrobials in different classes available to treat or prevent most human infections even if antimicrobial resistance to amoxicillin develops. In addition, it is considered more appropriate based on the susceptibility results of our study (Table 2).

Many patients in our study also received a combination of antimicrobials empirically, consistent with Australasian Infectious Diseases Advisory Panel (AIDAP) Antibiotic Prescribing Detailed Guidelines that suggested the use of parenteral amoxicillin, with or without either gentamicin or an injectable fluoroquinolone, and metronidazole, in severe cases of acute lower respiratory tract infection in dogs [22]. Anecdotally veterinary critical care specialists frequently perform daily airway cytology on ventilator patients, by taking a sterile swab from the secretions inside of a sterile endotracheal tube during daily tube change. Additionally, it is common clinical practice to broaden antimicrobial spectrum prior to the receipt of culture and susceptibility results, particularly by the addition of fluoroquinolones when intracellular rods are seen, the severity of septic suppurative inflammation is worsening, or the patient’s respiratory status is worsening.

Endotracheal aspirates were the predominant airway samples in this population, likely since they were more readily available and practical in cases with large volumes of exudate from the lower airways within the endotracheal tubes. Although the utility of ETA samples has not been compared to BAL in dogs or cats with pneumonia, a meta-analysis in critically ill human adults demonstrated a comparable sensitivity and specificity of ETA to BAL for the diagnosis of VAP, with histopathology as the reference standard [23]. Sensitivity was 75.7% (95% confidence interval [51.5–90.1]) for ETA and 71.1% (95% CI 49.9–85.9) for BAL, while specificity was 67.9% (95% CI 40.5–86.8) for ETA and 79.6% (95% CI 66.2–85.9) for BAL. As such, the use of either ETA or BAL is recommended for the diagnosis of VAP in several human guideline statements [24,25].

Most airway cultures were obtained within two days of intubation. Although the rationale for sample timing was not always evident in the medical records, it is likely that at least some were taken following the clinical progression of the disease, such as a regurgitation event or worsening of hypoxaemia. In the last 2–3 years it has become more of a standard of practice in authors’ institution to routinely perform airway cultures in intubated tick paralysis cases due to the anecdotal experience of high prevalence of aspiration pneumonia. While one case was classified as VAP, it is possible that the pneumonia was present earlier, but just not sampled or documented radiographically. As such, it is prudent that clinicians managing mechanically ventilated dogs and cats consider obtaining airway samples, by either ETA or BAL, as soon as there is clinical suspicion of pneumonia and patient stability allows.

Four of the six most prevalent microorganisms cultured in our study, *Staphylococcus* spp., *E. coli*, *Klebsiella* spp., and *Mycoplasma* spp., are similar to those in other large studies of dogs with aspiration pneumonia [16,26]. Interestingly however, *E. faecalis* and *E. faecium* were frequently isolated in our cases but do not feature heavily in the aforementioned publications. Nonetheless, these *Enterococcus* spp. are common commensal microbes of the gastrointestinal tract in dogs and cats, consistent with our clinical conclusions of aspiration pneumonia. Awareness of the potential for involvement of *Enterococcus* spp. in aspiration pneumonia in veterinary patients undergoing MV for tick paralysis is important given the high rates of intrinsic and acquired resistance in these organisms [27]. The identification of a significant survival benefit associated with the use of appropriate antimicrobials in our cases also supports the use of culture and susceptibility to guide antimicrobial therapy in ventilated animals. Of note, this survival benefit persisted regardless of the presence of MDR organisms.

There are several limitations in this study. Firstly, the sample size may have resulted in a type II error, including in the assessment of the effect size of antimicrobial appropriateness on survival to discharge. This sample size resulted in very wide confidence intervals in our estimates, where significance was achieved. The retrospective nature of this study meant that the rationale for clinician decision making was not always evident, precluding a full understanding of factors contributing to changes in antimicrobials and the main reason for MV for example. Additionally, most patients had just a single culture and susceptibility sample obtained during MV, which may have missed changes in the organisms involved, particularly in cases with repeated aspiration events. Furthermore, the use of empirical antimicrobials prior to collection of samples for culture is expected to have affected culture results and likely led to a higher incidence of MDR organisms than would have been present otherwise. Ideally, serial airway cytology would have been performed to aid in the interpretation of culture results and clinical course. In addition, cases of VAP may have been missed due to the timing of airway samples collected and the difficulty in identifying VAP in patients with preexisting aspiration pneumonia. Finally, there were limitations around the interpretation of thoracic radiographs. Firstly, interpretation of thoracic radiographs although standardised was not blinded and not all radiographs were available for interpretation by a board-certified radiologist. Additionally, atelectasis could not be ruled out in some cases; this could have been mitigated by standardised application of positive end expiratory pressure during acquisition of radiographs. 

Future directions to address some of these limitations could involve a larger, multi-centred prospective study with a standard protocol of airway sampling at intubation (prior to antimicrobials), and at 48 h thereafter, serial thoracic radiographs, daily airway cytology and arterial blood gases, and closer monitoring of aspiration events. Further research on strategies to reduce the risks of aspiration in dogs and cats with tick paralysis are also indicated.

## 5. Conclusions

This study is the first to describe a population of dogs and cats with positive airway cultures during mechanical ventilation for tick paralysis. Clinicians should have an index of suspicion for this complication and consider empirical antimicrobials appropriate for the likely organisms present and based on the severity of illness, pending the results of culture and susceptibility testing of airway samples. Culture and susceptibility can then be used to guide the escalation or de-escalation of antimicrobials to optimise outcomes while adhering to principles of antimicrobial stewardship. 

## Figures and Tables

**Table 1:** Classification and numbers of microorganisms cultured from the airways of dogs and cats with tick paralysis during mechanical ventilation, obtained by endotracheal aspirate (ETA) or bro nchoalveolar lavage (BAL).

Gram Positive Cocci (*n* = 25)	Gram Negative Rods (*n* = 26)	Others (*n* = 6)
*Enterococcus faecalis* (8—7 ETA, 1 BAL) *Staphylococcus pseudintermedius* (7—6 ETA, 1 BAL) *Staphylococcus felis* (1 ETA) ^#^ *Staphylococcus haemolyticus* (1—BAL) *Staphylococcus schleiferi* (1—BAL) *Staphylococcus* sp. * (1—ETA) *Enterococcus faecium* (3—2 ETA, 1 BAL) *Streptococcus* Group G (2—1 ETA, 1 BAL) *Streptococcus* Group C (1 BAL)	*Escherichia coli* (6—4 ETA, 2 BAL) *Klebsiella pneumoniae* (5—3 ETA, 2 BAL) *Klebsiella aerogenes* (2—1 ETA, 1 BAL) *Klebsiella oxytoca* (1 ETA) *Klebsiella* sp. (1 ETA) *Pseudomonas aeruginosa* (4 ETA) *Enterobacter cloacae* complex (2 ETA) *Enterobacter* sp. (1 ETA) *Acinetobacter baumannii* (1 BAL) *Aeromonas hydrophila* (1 ETA) *Serratia rubidaea* (1 ETA) *Proteus mirabilis* (1 ETA)	*Mycoplasma* sp. (3 ETA) ^#^ *Pasteurella* sp. (1 ETA) *Pasteurella multocida* (1 ETA) ^#^ *Candida* sp. (1 BAL)

* unspeciated coagulase negative *Staphylococcus* spp. ^#^ isolates from feline patients (1/3 *Mycoplasma* sp.).

**Table 2:** Summary of microbes cultured from the airways of dogs and cats undergoing mechanical ventilation for tick paralysis, and their antimicrobial susceptibility patterns. Proportions and percentages of bacterial isolates considered susceptible to given antimicrobials are displayed. Bacteria are classified as Gram-positive cocci, Gram-negative rods, or other. Antimicrobials are classified by class.

		Aminoglycosides		Beta-Lactams						Fluoroquinolones		Other					
	Number of Isolates	Amikacin	Gentamicin	Ampicillin/Amoxicillin	Amoxicillin-Clavulanate	Cephalexin	Cephalothin	Ceftazidime	Cefovecin	Enrofloxacin	Marbofloxacin	Chloramphenicol	Clindamycin	Erythromycin	Tetracycline	Doxycycline	Trimethoprim Sulfonamide
Gram positive cocci																	
*Enterococcus faecalis*	8	IR	IR	100 (8/8)	100 (8/8)	IR	IR	IR	IR	100 (8/8)	0 (0/5)	67 (2/3)	IR	100 (8/8)	50 (3/6)	67 (4/6)	IR
*S. pseudintermedius*	7	100 (1/1)	71 (5/7)	0 (0/7)	71 (5/7)	71 (5/7)	71 (5/7)	-	32 (3/7)	71 (5/7)	20 (1/5)	0 (0/2)	66 (4/6)	66 (4/6)	40 (2/5)	33 (1/3)	71 (5/7)
Other *Staphylococcus* species	4	-	75 (3/4)	50 (2/4)	50 (2/4)	50 (2/4)	50 (2/4)	100 (1/1)	50 (2/4)	50 (2/4)	0 (0/2)	100 (1/1)	50 (2/4)	67 (2/3)	100 (2/2)	100 (2/2)	50 (2/4)
*Enterococcus faecium*	3	IR	IR	0 (0/3)	0 (0/3)	IR	IR	IR	IR	0 (0/3)	0 (0/3)	100 (3/3)	IR	33 (1/3)	0 (0/1)	33 (1/3)	IR
*Streptococcus* species	3	-	0 (0/3)	100 (3/3)	100 (3/3)	100 (3/3)	100 (3/3)	-	-	0 (0/3)	100 (2/2)	-	100 (3/3)	100 (3/3)	50 (1/2)	50 (1/2)	100 (3/3)
Gram negative rods																	
*Escherichia coli*	6	-	100 (6/6)	67 (4/6)	67 (4/6)	67 (4/6)	67 (4/6)	-	67 (4/6)	100 (6/6)	100 (4/4)	-	100 (2/2)	0 (0/3)	-	80 (4/5)	67 (4/6)
*Klebsiella* species	9	-	100 (9/9)	IR	56 (5/9)	78 (7/9)	78 (7/9)	100 (2/2)	78 (7/9)	100 (9/9)	100 (3/3)	100 (1/1)	50 (1/2)	50 (1/2)	100 (6/6)	50 (2/4)	89 (8/9)
*Pseudomonas* *aeruginosa*	4	-	100 (4/4)	IR	IR	IR	IR	100 (4/4)	IR	25 (1/4)	-	IR	IR	IR	IR	IR	IR
*Enterobacter* species	3	-	100 (3/3)	IR	IR	IR	IR	IR	IR	33 (1/3)	0 (0/2)	100 (1/1)	-	-	100 (3/3)	100 (2/2)	33 (1/3)
*Acinetobacter* *baumannii*	1	-	100 (1/1)	IR	IR	0 (0/1)	0 (0/1)	IR	0 (0/1)	100 (1/1)	-	IR	-	-	100 (1/1)	-	IR
*Aeromonas hydrophila*	1	-	100 (1/1)	0 (0/1)	0 (0/1)	0 (0/1)	0 (0/1)	-	-	100 (1/1)	-	-	-	-	-	100 (1/1)	100 (1/1)
*Serratia rubidaea*	1	-	100 (1/1)	0 (0/1)	0 (0/1)	0 (0/1)	0 (0/1)	-	0 (0/1)	100 (1/1)	-	-	-	-	0 (0/1)	0 (0/1)	100 (1/1)
*Proteus mirabilis*	1	-	100 (1/1)	100 (1/1)	0 (0/1)	100 (1/1)	100 (1/1)	-	0 (0/1)	100 (1/1)	-	-	-	-	IR	0 (0/1)	100 (1/1)
Other																	
*Pasteurella multocida*	2	-	-	100 (2/2)	100 (2/2)	100 (2/2)	100 (2/2)	-	100 (2/2)	100 (2/2)	-	-	0 (0/1)	0 (0/1)	100 (1/1)	100 (1/1)	100 (2/2)

IR = Intrinsic resistance [17].

## Data Availability

The authors confirm that the data supporting the findings of this study are available within the article.
